# Genetic variants of PTGS2, TXA2R and TXAS1 are associated with carotid plaque vulnerability, platelet activation and TXA2 levels in ischemic stroke patients

**DOI:** 10.1371/journal.pone.0180704

**Published:** 2017-07-12

**Authors:** Xingyang Yi, Jing Lin, Hua Luo, Chun Wang, Yingying Liu

**Affiliations:** 1 Department of Neurology, People’s Hospital of Deyang City, Deyang, Sichuan, China; 2 Department of Neurology, the Third Affiliated Hospital of Wenzhou Medical University, Wenzhou, Zhejiang, China; 3 Department of Neurology, the Affiliated Hospital of Southwest Medical University, Luzhou, Sichuan, China; Boston University, UNITED STATES

## Abstract

Eicosanoids may play a role in ischemic stroke. However, the associations of variants in cyclooxygenase (COX) pathway genes and interaction among these variants with carotid plaque vulnerability are not fully understood. In present study, twelve variants in COX pathway genes were examined using matrix-assisted laser desorption ionization time-of-flight mass spectrometry method in 396 patients with ischemic stroke and 291 controls. Platelet aggregation, platelet-leukocyte aggregates, and urine 11-dehydrothromboxane B2 (11-dTxB2) were also measured. According to the results of carotid high-resolution B-mode ultrasound, the patients were stratified into the following groups [i.e., non-carotid plaque and carotid plaque. The carotid plaque was further classified into subgroups of echolucent plaque (ELP) and echogenic plaque (EGP)]. Additionally, gene-gene interactions were analyzed to assess whether there was any interactive role for assessed variants in affecting carotid plaque vulnerability, platelet activation and 11-dTxB2 levels. There were no significant differences in the frequencies of genotypes of the twelve variants between patients and controls. Among 396 patients, 294 cases (74.2%) had carotid plaques (106 had ELP, 188 had EGP). Frequency of *PTGS2* rs20417CC, *TXAS1* rs2267679TT, *TXAS1* rs41708TT, *PTGIS* rs5602CC, and *TXA2R* rs1131882TT genotype was significantly higher in patients with plaque compared with patients without plaque, or in patients with ELP compared with patients with EGP. 11-dTxB_2_ levels, platelet aggregation and platelet-leukocyte aggregates were significantly higher in patients with ELP compared with patients without plaque or with EGP. Multivariate logistic regression analysis revealed that *PTGS2* rs20417CC, *TXA2R* rs1131882TT, and high-risk interaction among variants in *PTGS2* rs20417, *TXA2R* rs1131882 and *TXAS1* rs41708 were independently associated with the risk of ELP after adjusting for confounding variables. The variants in COX pathway genes and the high-risk interactions among variants in *PTGS2* rs20417, *TXA2R* rs1131882 and *TXAS1* rs41708 were associated with high 11-dTxB2 and platelet activation, and independently associated with the risk of carotid plaque vulnerability. These variants might be potential markers for plaque instability.

## Introduction

Stroke is often caused by atherosclerotic plaque rupture [1]. Vulnerable lesions may block the blood flow to the brain by atherothrombosis of large cerebral arteries or as a result of emboli [2]. There has been an increasing awareness of the importance of carotid plaque vulnerability as a major risk factor for stroke. Plaque echogenicity as assessed by B-mode ultrasound has been found to reliably predict the content of soft tissue and the amount of calcification in carotid plaques [3]. Plaques that appear echolucent on B-mode ultrasound are lipid rich, whereas echogenic plaques have a higher content of dense fibrous tissue and calcification. Several cross-sectional studies have reported an association between echolucent or hypoechoic plaques and a history of TIAs and stroke [4]. Therefore, identifying novel risk factors for carotid plaque vulnerability is very important for preventing atherosclerosis and stroke [5].

Atherosclerosis is a complex inflammatory disorder. Differential risk of atherosclerosis in the population may reflect variations in genes that modulate the inflammatory response to oxidized lipids in the arterial walls [6]. A potential role for genes involved in inflammatory processes has been suggested in the pathogenesis of atherosclerosis [7,8]. Eicosanoids are arachidonic acid-derived lipid molecules, including lipoxygenase (LOX)-, cytochrome P450 (CYP)- and cyclooxygenase (COX)-derived metabolites that may play a key role in atherosclerosis [9]. It is well documented that arachidonic acid (AA) is readily metabolized by COX, LOX and CYP, generating prostanoids, leukotrienes, and epoxyeicosatrienoic acids, respectively [10]. Our previous studies have shown that CYP genetic polymorphisms and CYP metabolite levels are associated with carotid artery stenosis and plaque stability in ischemic stroke patients [11,12]. Genetic polymorphisms of LOX pathway genes increase susceptibility to ischemic stroke and are associated with atherothrombotic events in stroke patients [13]. However, our previous studies did not investigate the association of variants in COX pathway genes with ischemic stroke and carotid atherosclerosis.

The prostaglandins, prostacyclins (PGI), thromboxanes (TXA) are main eicosanoids. AA is metabolized by prostaglandin H synthase-1 and -2 (more commonly described as COX-1 and COX-2, respectively) to prostaglandin H2. Prostaglandin H2 is in turn metabolized to TXA2 by thromboxane synthase in platelets, to PGI2 by prostacyclin synthase in endothelial cells, and to prostaglandin E2 by prostaglandin E2 synthase in many different tissues [10]. PGI2 and TXA2 have opposite effects on blood flow and platelet activity and may play a key role in acute coronary syndromes and atherosclerosis [10]. TXA2 is a potent vasoconstrictor. PGI2 is a vasodilator that inhibits platelet activation and specifically limits the platelet response to TXA2. Binding of TXA2 to its receptor TXA2R may increase platelet aggregation. Therefore, TXA2R plays a key role in the pathogenesis of atherosclerosis and thrombosis [14]. The stable TXA2 metabolite 11-dehydrothromboxane B2 (11-dTxB2) reflects *in vivo* platelet activation and can be measured in plasma or urine. This metabolite is useful in monitoring platelet activity in patients. Several studies have shown that single nucleotide polymorphisms (SNPs) in the prostaglandin H synthase-1 gene (*PTGS1*), *PTGS2*, thromboxane synthase-1 gene (*TXAS1*), prostacyclin synthase gene (*PTGIS*) and thromboxane A2 receptor gene (*TXA2R*) are associated with cardiovascular disease or ischemic stroke [14–22]. However, the association of these genetic variants with carotid plaque vulnerability has not been well addressed.

In this study, we hypothesized that the variants in COX pathway genes and interaction among these variants might influence platelet activation and TXA2 levels, and were associated with carotid plaque vulnerability. Thus, we assessed 12 SNPs from COX pathway genes, platelet activation and TXA2 metabolite 11-dTxB2 in ischemic stroke patients and controls, and investigated the association of these genetic variants with carotid plaque vulnerability in Chinese population.

## Materials and methods

### Study population

This study protocol was reviewed and approved by the Ethics Committee of the People’s Hospital of Deyang City. Each of the participants provided informed consent (in Chinese language) before participating in this study. In most cases, the participants provided their written informed consent. For patients who could not read or write standard Chinese, the consent was verbal.

The detailed information of the study patients was described in our previous article [11]. We consecutively enrolled 396 ischemic stroke patients who received medical care in our hospitals between August 2010 and March 2013. Ischemic stroke was confirmed based on both clinical findings and the results of brain magnetic resonance imaging. Ischemic stroke in all cases was due to atherothrombotic (AT) and small artery disease (SAD), according to the Trial of ORG 10172 in the Acute Stroke Treatment (TOAST) classification system [23]. Exclusion criteria included: (1) carotid endarterectomy or stent implantation; (2) cardiogenic cerebral embolisms or ischemic stroke caused by unknown factors; (3) calcified plaques with acoustic shadow or occluded carotid artery, since reliably of determining their echogenicity was technically impossible; (4) family history of apoplexy or a previous history of strokes; (5) individuals declined to participate in the study.

The healthy volunteers who served as controls were selected from outpatients with no history of stroke as confirmed by medical history as well as physical and laboratory examinations at our center. They had no family history of stroke and were not genetically related to the stroke patients.

Data on various risk factors, including age, gender, current smoking, history of diabetes mellitus and hypertension, were recorded. Fasting blood samples were tested for blood sugar, hemoglobin A1c (HbA1c), triglycerides (TG), total plasma cholesterol (TC), low-density lipoprotein cholesterol (LDL-C), high-density lipoprotein cholesterol (HDL-C).

### Genotyping of SNPs

Whole blood (3 mL) was drawn from an arm vein into a sterile tube containing ethylenediaminetetraacetic acid (EDTA) and stored at -80°C until genotype analysis was performed. The 12 SNPs of the COX pathway genes were selected from the NCBI database (http://www.ncbi.nlm.nih.gov/SNP), according to the criteria: (1) the SNPs had been examined in previous studies [14–22]; (2) the SNPs lead to amino acid changes. Genotypes of the 12 SNPs were examined using matrix-assisted laser desorption ionization time-of-flight mass spectrometry methods according to our previous study [24]. In brief, each SNP gene possessed a specific genotype, with two amplification primers and one extension primer. The reaction mix was desalted by adding 6 mg of cation exchange resin (Sequenom Inc., San Diego, CA), mixed, and resuspended in 25 μL of water. Once the primer extension reaction was completed, the samples were spotted onto a 384-well spectroCHIP (Sequenom Inc., San Diego, CA) using a MassARRAY Nanodispenser (Sequenom Inc., San Diego, CA) and genotyped using the MALDI-TOF mass spectrometer. Genotyping was performed in real time with MassARRAY RT software, version 3.0.0.4, and analyzed using the MassARRAY Typer software, version 3.4 (Sequenom Inc., San Diego, CA).

### Carotid ultrasonography

Bilateral common and internal carotid arteries, as well as bifurcations, were examined for atherosclerotic plaque presence using a diagnostic ultrasound device (type 512, Acuson Sequoia Apparatus, 7.5-MHz probe, Berlin, Germany) in all patients, according to standard scanning and reading protocols [5]. Carotid plaque was defined as an endoluminal protrusion of at least 1.5 mm or a focal thickening >50% of the intima-media thickness relative to the adjacent wall segment [7,25]. According to the results, the patients were divided into two groups: carotid plaque and non-carotid plaque groups. Thereafter, types of plaques were defined by their echo structure [26]: class I, uniformly anechogenic; class II, predominantly hypoechogenic with >50% hypoechogenic area, class III, predominantly echogenic with >50% echogenic area; class IV, uniformly echogenic. Accordingly, patients in the carotid plaque group were divided in two groups: echolucent plaque (ELP; class I and class II) group and echogenic plaque (EGP; class III to class IV) group. Intraobserver and interobserver coefficients of variation for plaque echogenicity were 8.2% and 8.8%, respectively. The procedures for evaluating intraobserver and interobserver coefficients were performed as described in our previous studies [11].

### Measurement of platelet aggregation and platelet-leukocyte aggregates

Venous blood (6 mL) was drawn from an antecubital vein in each patient on admission. Platelet aggregation was measured by light transmittance aggregometry (LTA) using our previously described procedures [27]. Whole-blood specimens were centrifuged at 200×g for 10 min to obtain platelet-rich plasma (PRP). Platelet-poor plasma (PPP) was obtained from the remaining specimens by centrifugation at 4000×g for 10 min. Classical OPA in PRP ad modum Born was performed using a PAP-4D aggregometer (Bio/Data Corporation, Alpha Laboratories Limited, Horsham, USA). Platelet aggregation was recorded as changes in light transmission. The recorder was adjusted to make sure that the difference in light transmission between PRP and PPP was 100%. The results of OPA are presented as the amplitude of light transmittance at five minutes after addition of the agonist 0.5 mM AA and 10 μM ADP (Helena Laboratories, Beaumont, TX, USA). Platelet-leukocyte aggregates were measured by FC 500 MPC flow cytometry (Beckman Coulter Ltd, Krefeld, Germany), and we used direct fluorescent markers (all commercially available; Coulter Immunotech, Krefeld, Germany). The procedures were performed as described in our previous studies [28]. In brief, Whole blood was diluted 1:10 with warmed HEPES buffer. Two aliquots of 50 mL were incubated with CD61-phycoerythrin (an activation-independent subunit of the GP IIb/IIIa complex) to immunologically identify all platelets. Simultaneously, in a one-step procedure, the sample for measuring platelet activation was stained with anti-CD62P. The other sample was double-stained with the pan leukocytic marker CD45 to identify leukocytes. After incubation for 5 min, the process was stopped using cold buffer and immediately followed by flow cytometry.

### Urine 11-dTxB_2_ levels

Urine samples (5 mL) were collected in each patient on admission. 11-dTxB2 levels were measured in urine samples using a commercially available ELISA kit (11-Dehydro-thromboxane B2 EIA Kit, Cayman Chemical, San Antonio, TX, USA) following the manufacturer’s instructions. All urine samples were assayed in duplicate, and the mean intra-assay coefficient of variation (CV, %) was determined. The procedures were performed as described in our previous studies [29].

### Statistical analysis

Based on a suggested sample size requirement for detecting gene-gene interactions [30], we expected that our sample size of 180 patients with EGP and 100 patients with ELP would sufficiently provide 80% power at the 5% significance level calculated using three genetic models: the additive model, the dominant model, and the recessive model.

All statistical analyses were performed using SPSS 16.0 (SPSS Inc., Chicago, IL, USA). Categorical variables are presented as frequencies and percentages, and differences among patients with ELP, EGP and Non- plaque, or between stroke patients and controls were evaluated using χ2-test, or Fisher’s exact tests. Continuous variables are expressed as mean ± Standard Deviation (SD), and differences among patients with ELP, EGP and Non- plaque were evaluated using analysis of variance (ANOVA) followed by Student-Newman-Keuls test. Evaluating Hardy–Weinberg equilibrium were analyzed by χ2-test. Difference of genotype frequencies among patients with ELP, EGP and Non- plaque, or between stroke patients and controls was compared by χ2-test. Differences of 11-dTxB_2_, platelet aggregation, and platelet-leukocyte aggregates among genotypes were compared using analysis of variance (ANOVA) followed by Student-Newman-Keuls test.

Gene-gene interaction was assessed using the generalized multifactor dimensionality reduction (GMDR) method, as described in our previous studies [24]. The GMDR computed the maximum likelihood estimates and the scores of all individuals under the null hypothesis. The GMDR v0.7 program was used in this study (www.healthsystem.virginia.edu/internet/addiction-genomics/Software) [24,31]. A cumulative score was then calculated within each multifactor cell, which was labeled either as high-risk if the average score met or exceeded a pre-assigned threshold of zero, or as low-risk if the score was less than zero. An exhaustive search of all possible one- to ten-locus models was performed for all variants. The model with the minimum prediction error, the maximum cross-validation consistency score, and a *P* value of 0.05 or less (derived automatically from the sign test in the GMDR software) was considered as the best model. This model was then confirmed by a permutation test implemented in the GMDR software. Furthermore, multivariate logistic regression analysis was performed to adjust covariate risk factors using variables with *P* values < 0.05 in univariate analysis to assess the independent contribution of the SNPs and these gene-gene interactions on carotid plaque vulnerability, and odds ratio (OR) with 95% confidence intervals (CI) were calculated. All tests were two sided, and *P* value less than 0.05 were considered statistically significant.

## Results

### Comparison of clinical characteristics and genotype distributions between patients and controls

As expected, the stroke patients had a higher prevalence of risk factors, including a history of hypertension (*P* < 0.001) and diabetes mellitus (*P* = 0.006). However, differences in other conventional risk factors including age, gender, smoking, body mass index, and drug treatments were not significantly different between patients and controls (S1 Table). The genotype distributions of the 12 variants in this study did not deviate from the Hardy-Weinberg Equilibrium model (*P* > 0.05). There were no statistically significant differences in the frequencies of the genotypes of the 12 variants between the two groups (S1 Table).

### Characteristics of patients

Among these 396 patients, 276 cases (69.7%) were AT stroke, and 120 cases (30.3%) were SAD stroke, 294 cases (74.2%) had plaques (106 had ELP, 188 had EGP). The clinical characteristics of the patients are presented in Table 1. Hypertension, diabetes mellitus and AT stroke were significantly more frequent in patients with ELP or EGP than in patients without plaque. TC levels were also higher in patients with ELP or EGP than in patients without plaque. Hypertension and diabetes mellitus were significantly more frequent in patients with ELP than in patients with EGP. However, differences in other conventional risk factors did not reach statistical significance among the three groups.

**Table 1:** Clinical characteristics of patients with or without carotid plaques.

Characteristics	ELP (n = 106)	EGP (n = 188)	Non- plaque (n = 102)	*P* value
Age (years)	68.62± 10.84	68.13 ± 11.92	67.94 ± 11.86	0.535
Men (n, %)	62 (58.49)	112 (59.57)	61 (59.80)	0.912
Diabetes mellitus (n, %)	50 (47.17)	64 (34.04)	24 (23.53)	0.008
Hypertension (n, %)	95 (89.62)	140 (74.47)	52 (50.98)	<0.001
Previous MI (n, %)	1 (0.94)	2 (1.06)	2 (1.96)	0.986
Current smoking (n, %)	45 (42.45)	75 (39.89)	40 (39.22)	0.898
Body mass index (kg/m^2^)	24.09 ± 2.51	24.02 ± 2.59	23.96 ± 2.64	0.582
TC (mM)	5.62 ± 1.38	5.53± 1.32	5.36 ± 1.21	0.016
LDL-C (mM)	3.13 ± 1.23	2.93 ± 1.20	2.99 ± 1.19	0.183
HDL-C(mM)	1.22 ± 0.47	1.22 ± 0.53	1.23 ± 0.51	0.782
TG (mM)	1.92 ± 1.09	1.87 ± 1.11	1.83 ± 1.02	0.512
Fasting blood-glucose (mM)	7.08 ± 2.14	6.99±2.09	6.86 ± 2.35	0.315
HbA1c (%)	6.22 ± 1.38	6.11±1.35	6.04 ± 1.49	0.136
Previous or ongoing drug treatments (n, %)				
Antihypertensive drugs	32 (30.19)	57 (30.32)	32 (31.37)	0.893
Hypoglycemic drugs	29 (27.36)	47 (25.00)	19 (18.63)	0.127
Statins	14 (13.21)	25 (13.29)	12 (11.76)	0.336
Antiplatelet drugs	21 (19.81)	39 (20.75)	23 (22.55)	0.875
Stroke subtype (n, %)				
AT stroke	78 (73.58)	135 (71.81)	63 (61.76)	0.046
SAD stroke	28 (26.42)	53 (28.19)	39 (38.24)	-

MI, myocardial infarction; TC, total cholesterol; LDL-C, low-density lipoprotein cholesterol; HDL-C, high-density lipoprotein cholesterol; TG, triglycerides; HbA1c, Hemoglobin A1C; ELP, echolucent plaque; EGP, echogenic plaque; AT, atherothrombotic; SAD, small artery disease.

### Genotype distributions, 11-dTxB2, platelet aggregation and platelet-leukocyte aggregates comparison in patients with or without plaque

Frequency of rs20417CC, rs2267679TT, rs41708TT, rs5602CC, and rs1131882TT genotype was significantly higher in patients with plaque compared with patients without plaque, or in patients with ELP compared with patients with EGP (Table 2). 11-dTxB_2_ levels, platelet aggregation and platelet-leukocyte aggregates were significantly higher in patients with ELP compared with patients without plaque or with EGP (Table 2).

**Table 2:** Genotype distribution, 11-dTxB_2_, platelet aggregation and platelet-leukocyte aggregates comparison among the three groups (*n*, %).

	ELP (n = 106)	EGP (n = 188)	Non- plaque (n = 102)	*P* value
*PTGS1* (rs1236913)				
CC	102 (96.2)	184 (99.1)	100 (98.0)	0.936
CT	4 (3.8)	4 (2.1)	2 (2.0)	
TT	0	0	0	
*PTGS1* (rs3842787)				
CC	82 (77.4)	142 (75.5)	80 (78.4)	0.983
CT	16 (15.1)	31 (16.5)	15 (14.7)	
TT	8 (7.5)	15 (8.0)	7 (6.9)	
*PTGS2* (rs689466)				
AA	30 (28.3)	55 (29.3)	27 (26.5)	0.734
AG	53 (50.0)	91 (48.4)	50 (49.0)	
GG	23 (21.7)	42 (22.3)	25 (24.5)	
*PTGS2* (rs20417)				
GG	57 (53.8)	116 (61.7)	72 (70.6)	0.021
GC	26 (24.5)	43 (22.9)	24 (23.5)	
CC	23 (21.7)	29 (15.4)	6 (5.9)	
*TXAS1* (rs194149)				
AA	17 (16.0)	33 (17.5)	15 (14.7)	0.916
AG	53 (50.0)	90 (47.9)	54 (52.9)	
GG	36 (34.0)	65 (34.6)	33 (32.4)	
*TXAS1* (rs2267679)				
CC	2 (1.9)	8 (4.3)	1 (0.9)	<0.001
CT	14 (13.2)	42 (22.3)	35 (34.3)	
TT	90 (84.9)	138 (73.4)	66 (64.7)	
*TXAS1* (rs41708)				
GG	53 (50.0)	114 (60.6)	72 (70.6)	<0.001
GT	30 (28.3)	55 (29.3)	25 (24.5)	
TT	23 (21.7)	19 (10.1)	5 (4.9)	
*PTGIS* (rs45498106)				
GG	106 (100)	188 (100)	102 (100)	--
*PTGIS* (rs5602)				
TT	32 (30.2)	71 (37.8)	47 (46.1)	0.008
TC	47 (44.3)	89 (47.3)	49 (48.0)	
CC	27 (25.5)	28 (14.9)	6 (5.9)	
*PTGIS* (rs5629)				
AA	7 (6.6)	13 (6.9)	6 (5.9)	0.962
AC	36 (34.0)	62 (33.0)	31(30.4)	
CC	63 (59.4)	113 (60.1)	65 (63.7)	
*PTGES* (rs6478818)				
AA	91 (85.8)	165 (87.8)	88 (86.3)	0.968
AG	13 (12.3)	20 (10.6)	13 (12.7)	
GG	2 (1.9)	3 (1.6)	1 (1.0)	
*TXA2R* (rs1131882)				
CC	28 (26.4)	65 (34.6)	42 (41.2)	0.009
CT	47 (44.3)	87 (46.3)	50 (49.0)	
TT	31 (29.2)	36 (19.1)	10 (9.8)	
11-dTxB_2_ (ng/mmol creat)	204.6 ± 73.2	158.9 ± 61.3	129.7 ± 57.6	<0.001
Platelet aggregation (%)				
AA-induced	91.2 ± 11.4	86.9 ± 10.4	84.6 ± 11.7	0.002
ADP-induced	89.7 ± 12.1	86.1 ± 11.5	84.7 ± 10.8	0.008
Platelet-leukocyte aggregates (%)				
Leukocyte	28.1 ± 6.7	23.8 ± 5.4	22.8 ± 7.2	<0.001
Neutrophil	27.2 ± 6.8	22.9 ± 7.1	22.2 ± 6.4	<0.001
Monocyte	26.9 ± 5.5	22.3 ± 6.3	21.8 ± 4.7	<0.001
Lymphocyte	26.4 ± 5.6	22.4 ± 7.5	21.8 ± 5.8	<0.001

11-dTxB_2_, 11-dehydro-thromboxane B_2_; Creat, creatine; AA, arachidonic acid; ADP, adenosine diphosphate.

### Gene-gene interaction and carotid plaque vulnerability

For carotid plaque vulnerability, high-order interactions were investigated using the GMDR method. With covariate adjustments, the best model for ELP was rs20417, rs1131882 and rs41708, which scored 10/10 for cross-validation consistency and 9/10 for the sign test (*P* = 0.017, Table 3). The one-locus model was also computed for each variant. The significance of the interactions was further confirmed by a permutation test (*P* = 0.032), suggesting a synergistic effect of the three variants contribution to risk of carotid plaque vulnerability.

**Table 3:** Comparison of the best models, prediction accuracies, cross-validation consistencies, and *P* values for echolucent plaque identified by GMDR.

Best model*	Training balanced accuracy	Testing balanced accuracy	Cross-validationconsistency	Sign test (*P*)
1	0.468	0.612	5/10	7 (0.463)
1, 2	0.547	0.622	9/10	9 (0.268)
1, 2, 3	0.697	0.663	10/10	9 (0.017)
1, 2, 3, 4	0.622	0.602	8/10	8 (0.314)
1, 2, 3, 4, 5	0.575	0.496	7/10	7 (0.613)
1, 2, 3, 4, 5, 6	0.614	0.521	6/10	8 (0.542)
1, 2, 3, 4, 5, 6, 7	0.527	0.466	7/10	5 (0.725)
1, 2, 3, 4, 5, 6, 7, 8	0.645	0.562	8/10	6 (0.313)
1, 2, 3, 4, 5, 6, 7, 8, 9	0.602	0.554	7/10	7 (0.685)
1, 2, 3, 4, 5, 6, 7, 8, 9, 10	0.578	0.564	6/10	5 (0.782)
1, 2, 3, 4, 5, 6, 7, 8, 9, 10,11	0.485	0.643	8/10	6 (0.325)
1, 2, 3, 4, 5, 6, 7, 8, 9, 10,11,12	0.511	0.572	6/10	7 (0.413)

*rs20417, rs1131882, rs41708, rs5602, rs1236913, rs3842787, rs689466, rs194149, rs2267679, rs45498106, rs5629, rs6478818 are symbolized as 1–12, respectively.

GMDR, generalized multifactor dimensionality reduction.

Then, the associations between different combinations of genotypes and ELP were compared with wild-type genotypes rs20417GG, rs1131882CC and rs41708GG. The three interactions making large contributions to this model were among rs20417CC, rs1131882TT and rs41708TT; rs20417CC, rs1131882CT and rs41708GT; and rs20417CC, rs1131882TT and rs41708GT/TT (Table 4). The three combinations of the genotypes were defined as high-risk interactions. The other combinations of genotypes among rs20417, rs1131882 and rs41708 did not reach the cut-off significance level of 0.05, and were defined as low-risk interactions (Table 4).

**Table 4:** Associations between genotype combinations and echolucent plaque.

rs20417	GG	CC	CC	CC	GC	CC	CC, GC	CC, GC
rs1131882	CC	TT	CT	TT	CT	TT, CT	TT	TT, CT
rs41708	GG	TT	GT	GT,TT	GT	TT	TT	TT, GT
OR	1 *	2.72	2.18	2.04	1.21	1.02	1.03	1.12
95% CI	-	1.31–8.27	1.14–6.65	1.02–4.97	0.89–2.68	0.68–1.99	0.71–1.94	0.94–2.01
P value	-	0.003	0.019	0.031	0.224	0.546	0.612	0.336

* The low-risk genotype for each genetic factor was used as the reference OR. OR, odds ratio; CI, confidence interva.

Furthermore, the relative risk of ELP conferred by the combinations of variants in the three genes was considered as an interactive variable, with high-risk interactions assigned as one and low-risk interactions assigned as zero. Multivariate logistic regression analysis revealed that rs20417CC, rs1131882TT, and the high-risk interaction among variants in rs20417, rs1131882 and rs41708 were independently associated with the risk of ELP after adjusting for hypertension, diabetes mellitus, AT stroke and TC (Table 5).

**Table 5:** Multivariate analysis of the major risk factors for echolucent plaques.

Risk factor	*OR*	95% CI	*P* value
Hypertension	1.88	1.02–3.42	0.043
Diabetes mellitus	0.94	0.75–1.76	0.473
AT stroke	0.72	0.63–1.25	0.823
TC	0.74	0.65–1.46	0.578
rs20417CC	1.94	1.02–3.85	0.035
rs2267679TT	1.16	0.95–2.14	0.156
rs41708TT	1.32	0.98–2.96	0.106
rs5602CC	1.05	0.89–1.96	0.268
rs1131882TT	2.02	1.13–5.32	0.026
High-risk interactions	2.42	1.36–7.64	0.005

OR, odds ratios; CI, confidence interval; AT, atherothrombotic; TC, total cholesterol.

### Effect of genotypes on platelet activation and urinary 11-dTxB_2_ levels

The platelet aggregation induced by AA or ADP and platelet–leukocyte aggregates on admission were higher in patients carrying rs20417CC than GG/GC, rs2267679TT than CC/CT, rs41708TT than GG/GT, rs5602CC than TT/TC, and rs1131882TT than CC/CT, and high-risk interactive genotypes than low-risk interactive genotypes among rs20417, rs1131882 and rs41708 (Table 6). However, there was no significant difference of the platelet aggregation and platelet-leukocyte aggregates among the genotypes of other 7 variants. Urinary 11-dTxB_2_ levels were also higher in patients carrying rs20417CC than GG/GC, rs2267679TT than CC/CT, rs41708TT than GG/GT, and high-risk interactive genotypes than low-risk interactive genotypes among rs20417, rs1131882 and rs41708 (Table 6). However, there was no significant difference of the urinary 11-dTxB_2_ levels among the genotypes of other 9 variants.

**Table 6:** Comparison of 11-dTxB_2_, platelet aggregation and platelet-leukocyte aggregates among genotypes.

1	11-dTxB_2_ Platelet aggregation (%) (ng/mmol creat) AA-induced ADP-induced			Platelet-leukocyte aggregates (%) Leukocyte Neutrophil Monocyte Lymphocyte			
rs20417							
GG (n = 245)	128.3 ± 54.5	84.2 ± 14.7	84.6 ± 13.6	23.3 ± 5.6	22.7 ± 4.9	23.1 ± 5.8	22.7 ± 4.7
GC (n = 93)	149.8 ± 60.5	85.1 ± 10.5	83.9 ± 11.2	22.8 ± 5.2	23.0 ± 4.3	22.6 ± 4.2	23.4 ± 3.8
CC (n = 58)	208.4 ± 72.4	90.2 ± 10.1	89.7 ± 9.8	27.7 ± 4.4	26.9 ± 3.6	26.7 ± 4.6	26.8 ± 4.5
*P* value	<0.001	0.011	<0.001	<0.001	<0.001	<0.001	<0.001
rs2267679							
CC (n = 11)	118.6 ± 32.4	83.2 ± 8.4	83.1 ± 11.7	22.8 ± 3.4	23.1 ± 2.8	22.7 ± 3.2	23.2 ± 2.7
CT (n = 91)	138.8 ± 56.7	84.3 ± 9.6	84.2 ± 10.9	22.9 ± 4.1	22.9 ± 4.2	23.4 ± 4.7	23.3 ± 4.8
TT (n = 294)	212.4 ± 80.8	89.9 ± 14.3	89.7 ± 13.3	26.6 ± 5.2	26.8 ± 5.7	26.8 ± 6.3	26.7 ± 5.5
*P* value	<0.001	0.017	0.036	<0.001	<0.001	<0.001	<0.001
rs41708							
GG (n = 239)	138.7 ± 81.5	85.2 ± 14.8	84.2 ± 13.6	22.7 ± 5.8	22.6 ± 5.7	22.5 ± 5.7	22.8 ± 4.9
GT (n = 110)	139.7 ± 61.4	84.3 ± 11.3	83.7 ± 12.2	22.8 ± 4.7	22.2 ± 3.8	23.1 ± 4.5	23.5 ± 4.1
TT (n = 47)	209.4 ± 48.7	90.7 ± 9.3	91.2 ± 10.2	27.2 ± 6.3	26.9 ± 4.5	27.2 ± 4.6	26.9 ± 3.2
*P* value	<0.001	<0.001	<0.001	<0.001	<0.001	<0.001	<0.001
rs5602							
TT (n = 150)	154.7 ± 63.4	84.6 ± 12.4	84.4 ± 13.4	22.6 ± 4.4	22.5 ± 4.5	23.1 ± 3.9	22.6 ± 5.8
TC (n = 185)	163.2 ± 71.4	85.1 ± 13.2	84.6 ± 12.6	23.4 ± 4.6	23.2 ± 4.3	22.5 ± 4.9	22.2 ± 5.3
CC (n = 61)	178.9 ± 60.7	88.8 ± 10.3	89.4 ± 10.6	27.2 ± 5.2	27.1 ± 4.3	27.1 ± 3.8	26.9 ± 4.2
*P* value	0.795	0.024	0.007	<0.001	<0.001	<0.001	<0.001
rs1131882							
CC (n = 135)	161.2 ± 78.7	85.4 ± 12.2	84.8 ± 12.4	22,3 ± 5.5	22.8 ± 5.6	23.1 ± 5.2	22.7 ± 6.4
CT (n = 184)	157.2 ± 81.5	84.9 ± 13.6	85.1 ± 11.9	22.6 ± 3.9	23.2 ± 4.4	22.8 ± 4.2	22.2 ± 5.3
TT (n = 77)	182.7 ± 54.7	90.2± 11.2	89.9 ± 10.4	26.9 ± 3.8	27.1 ± 3.4	27.2 ± 5.8	26.9 ± 4.8
*P* value	0.862	0.005	0.004	<0.001	<0.001	<0.001	<0.001
High-risk interactions							
Yes (n = 86)	232.5 ± 56.7	90.6 ± 11.8	89.9 ± 12.7	26.7 ± 5.6	27.1 ± 6.2	27.3 ± 4.8	26.9 ± 5.6
No (n = 310)	131.4 ± 88.6	85.6 ± 14.8	85.4 ± 13.2	23.2 ± 6.9	23.2 ± 5.3	23.8 ± 7.2	22.3 ± 3.9
*P* value	<0.001	0.002	0.006	<0.001	<0.001	<0.001	<0.001

AA, arachidonic acid; ADP, adenosine diphosphate. Creat, creatine; 11-dTxB_2_, 11-dehydro-thromboxane B_2_.

## Discussion

In the present study we did not find significant differences in the frequencies of the genotypes of the 12 variants in COX pathway genes between patients and healthy controls. However, we found that the variants of *PTGS2* rs20417, *TXA2R* rs1131882 and *TXAS1* rs41708 in COX pathway genes, and high-risk interactions among the three variants were associated with high 11-dTxB_2_ and platelet activation, and independently associated with the risk of carotid plaque vulnerability.

Several studies have investigated the association of a functional polymorphism of *PTGS2* rs20417 and *TXA2R* rs1131882 with the risk of cardiovascular disease or ischemic stroke [14–18, 21,22]. In the Atherosclerosis Risk in Communities (ARIC) study and one other study, the C allele of *PTGS2* rs20417 was reported to be associated with higher risk of stroke in African Americans or Brazilian population [16,32]. In contrast, the C allele was associated with lower risks of myocardial infarction, ischemic stroke and carotid intima-media thickness in an Italian population [15,33]. Binding of TXA2 to TXA2R may modulate thrombosis and play a vital role in the pathogenesis of ischemic stroke. However, we did not find significant differences in the frequencies of the genotypes of the 12 variants between patients and controls in this study. The results were inconsistent with above previous studies. There could be numerous potential explanations for the differences in the findings of the present study. The first reason may be attributed to the racial differences in the population of ischemic stroke patients being investigated. Indeed, significant SNP variations have been noted among different ethnic groups. A second explanation may be the complexity of ischemic stroke etiology itself. As a matter of fact, it is highly likely that the pathogenesis of ischemic stroke requires several variations, each with minor effects and potentially undetectable effects [24]. Therefore, a linkage analysis, which is used to investigate single-gene disorders, seems unsuitable for genetic studies on ischemic stroke. Third, may be due to the limited sample size and the one-center design of this study. Thus, our findings needs to be confirmed in large sample size and multi-center studies. Finally, environmental or lifestyle interaction with genes also may be one of the reasons why controls and patients do not show difference in the COX gene SNP frequencies.

Up to date, few studies to investigate the association between these variations in COX pathway genes and carotid plaque vulnerability. Key observations in the present study were identified via the GMDR approach. We detected interesting synergistic effects of a gene variant-gene variant interactions on carotid plaque vulnerability, and may affect platelet function and 11-dTxB_2_ levels. Variants in rs20417, rs1131882 and rs41708 were identified to interact together to influence the risk of carotid plaque vulnerability. Compared with wild-type genotype combination of rs20417GG, rs1131882CC and rs41708GG, there was a 2.72-fold increased risk for carotid plaque vulnerability in individuals with a combined genotype of rs20417CC, rs1131882TT and rs41708TT. These findings are very interesting.

Despite our experiments, the nature of the interactions among the three gene variants is unclear. Atherosclerosis development is associated with chronic inflammatory conditions. Eicosanoids, including PGI and TXA, are lipid mediators that may play a role in inflammatory processes and atherosclerosis [9]. Our current study demonstrated that urinary 11-dTxB_2_ levels, the platelet aggregation and platelet-leukocyte aggregates were significantly higher in patients with high-risk interactive genotypes than patients with low-risk interactive genotypes. One possible explanation for the three variants interactions is that the three variants encode for the enzymes and TXA2R that participate in AA metabolism, and impaire eicosanoids equilibrium. PGI2 and TXA2 have opposite effects on blood flow and platelet activity. TXA2 is a potent platelet activator and vasoconstrictor, and may play a key role in acute coronary syndromes and atherosclerosis [34]. PGI2 is a vasodilator that inhibits platelet activation and specifically limits the platelet response to TXA2. One experimental study described that reciprocal alterations in PGI2 and TXA2 may contribute to impaired angiogenesis [35]. PGI2/TXA2 imbalance could play a role in vascular disorders and cerebral blood flow, and contribute to cerebral ischemia/reperfusion injury [36,37]. TXAS1 and TXA2R are key components in TXA2 function [22]. The binding of TXA2 to TXA2R is crucial for platelet activation. Therefore, TXA2R plays a central role in the pathogenesis of atherosclerosis and thrombosis [14]. Several lines of evidence suggest a critical role of COX-2 expression in ischemic stroke, atherosclerosis and cancer, and selective COX-2 inhibitors may represent novel chemopreventive tools [38,39]. Lower COX-2 expression also reduces the extent of ischemic brain injury after a cerebral infarct [40]. The *PTGS2* rs20417, *TXA2R* rs1131882 and *TXAS1* rs41708 encode PTGS2, TXAS1 synthase and TXA2R, respectively. Numerous genetic polymorphisms in *PTGS2* have been identified and characterized [41]. The C allele of *PTGS2* rs20417 was reported to be associated with COX-2 activity and higher risk of stroke in African Americans [16,32]. Up to date, the association of *TBXAS1* variation with cardiovascular disease and ischemic stroke has not been well addressed. Our recent study showed that *TXAS1* rs41708 polymorphisms were independent risk factors for symptomatic carotid artery or intracranial arterial stenosis [42]. Variants of *TXA2R* led to increased ligand binding-induced intracellular calcium influx and fibrinogen-integrin conjugation, may affect platelet function and the risk of developing cerebral infarction [14,21,22]. Thus, the interactions among *PTGS2* rs20417, *TXA2R* rs1131882 and *TXAS1* rs41708 may affect the activity of PTGS2 and TXAS1 synthase, as well as binding of TXA2 to TXA2R, and provide these individuals with lower PGI2 and higher TXA2 than those without this particular gene variant interaction, thereby increasing the risk for carotid plaque vulnerability. However, further well designed studies are warranted to replicate this finding.

There are several potential limitations in the present study. First, our study was focused on Han Chinese population, and a single hospital study with limited sample size. Our findings thus need to be confirmed in multi-center studies with large sample sizes and other ethnicities. Second, although we demonstrated the association of variants in COX pathway genes with TXA2 metabolite 11-dTxB_2_, platelet aggregation and platelet-leukocyte aggregates, the PGI2 levels, COX activity, TXAS and TXAR expression were not measured in this study. In future studies, we will measure plasma PGI2 levels, COX activity, TXAS and TXAR expression, and to validate the correlations of these genetic polymorphisms with the PGI2 levels, COX activity, TXAS and TXAR expression. Third, although this study examined the role of several known important COX pathway genes, other known and unknown genes were not captured. Thus, future studies involving a larger set of genetic variants should be conducted to elucidate the full extent of gene-gene interaction effects on carotid plaque vulnerability pathogenesis. Forth, we main aim of the present study was to investigate the association of genetic variants in COX pathway genes with carotid plaque vulnerability in ischemic stroke patients. Many factors may affect the accuracy of carotid stenosis degree using carotid ultrasonography. Thus, we did not investigate the relations between these variants and carotid artery stenosis in this study. We will investigate the association of these variants with carotid artery stenosis in future. Finally, anti-inflammation drugs (i.e. statins) may affect platelet activation and carotid plaque characteristics. However, our result did not show the association of previous statins treatment with carotid plaque characteristics, the low proportion of statins treatment before stroke may be an important reason in china; thus, the study of larger sample is necessary to investigate the effect of statins on carotid plaque stability in future.

## Conclusions

The variants in COX pathway genes and the high-risk interactions among variants in *PTGS2* rs20417, *TXA2R* rs1131882 and *TXAS1* rs41708 were associated with high 11-dTxB2 and platelet activation, and independently associated with the risk of carotid plaque vulnerability. These variants might be potential markers for plaque instability. The combinational analysis used in this study may provide further insight into the complex genetic risk of carotid plaque vulnerability. However, further studies are needed to validate our findings.

## Supporting information

S1 Table Genotype and clinical characteristics comparison between patients and controls (*n*, %). (DOCX)
