# Supplementary material for: Genetic variants of PTGS2, TXA2R and TXAS1 are associated with carotid plaque vulnerability, platelet activation and TXA2 levels in ischemic stroke patients
Source: PLoS One. 2017 Jul 12;12(7):e0180704. doi: 10.1371/journal.pone.0180704 (PMC5507514; doi:10.1371/journal.pone.0180704)
Supplement: S1 Table — (DOCX) [file pone.0180704.s001.docx]

**S1 Table. Genotype and clinical characteristics comparison between patients and controls (*n,* %)**

|  | Stroke patients  (n = 396) | Controls  (n = 291) | *P* value |  |
| --- | --- | --- | --- | --- |
| *PTGS1* (rs1236913)  CC  CT  TT | 386 (97.5)  10 (2.5)  0 | 288 (99.0)  3 (1.0)  0 | 0.792 |  |
| *PTGS1* (rs3842787)  CC  CT  TT | 304 (76.8)  62 (15.6)  30 (7.6) | 229 (78.7)  42 (14.4)  20 (6.9) | 0.834 |  |
| *PTGS2* (rs689466)  AA  AG  GG | 112 (28.3)  194 (49.0)  90 (22.7) | 72(24.7)  143(49.1)  76(26.1) | 0.256 |  |
| *PTGS2* (rs20417)  GG  GC  CC | 245 (61.9)  93 (23.5)  58 (14.6) | 196 (67.4)  67 (23.0)  28 (9.6) | 0.186 |  |
| *TXAS1* (rs194149)  AA  AG  GG | 65 (16.4)  197 (49.7)  134 (33.8) | 40(13.7)  164(56.4)  87(29.9) | 0.193 |  |
| *TXAS1* (rs2267679)  CC  CT  TT | 11 (2.8)  91 (23.0)  294 (74.2) | 1(0. 3)  47(16.2)  243(83.5) | 0.106 |  |
| *TXAS1* (rs41708)  GG  GT  TT | 239 (60.4)  110 (27.8)  47 (11.9) | 177(60.8)  92(31.6)  22(7.6) | 0.764 |  |
| *PTGIS* (rs45498106)  GG | 396 (100) | 291 (100) | -- |  |
| *PTGIS* (rs5602)  TT  TC  CC | 150 (37.9)  185 (46.7)  61 (15.4) | 125(43.0)  131(45.0)  35(12.0) | 0.135 |  |
| *PTGIS* (rs5629)  AA  AC  CC | 26 (6.6)  129 (32.6)  241 (60.8) | 18(6.2)  91(31.3)  182(62.5) | 0.842 |  |
| *PTGES* (rs6478818)  AA  AG  GG | 344 (86.9)  46 (11.6)  6 (1.5) | 248(85.2)  40(13.8)  3(1.0) | 0.856 |  |
| *TXA2R* (rs1131882)  CC  CT  TT | 135 (34.1)  184 (46.5)  77 (19.4) | 65 (34.6)  87 (46.3)  36 (19.1) | 0.978 |  |
| Age (years) | 68.4 ± 11.8 | 66.9 ± 10.9 | 0.083 |  |
| Men (n, %) | 235 (59.3) | 165 (56.7) | 0.473 |  |
| Diabetes mellitus (n, %) | 138 (34.8) | 73 (25.1) | 0.006 |  |
| Hypertension (n, %) | 287 (72.5) | 130 (44.7) | <0.001 |  |
| Current smoking (n, %) | 160 (40.4) | 118 (40.5) | 0.998 |  |
| Body mass index (kg/m^2^) | 24.1 ± 2.3 | 23.9 ± 2.5 | 0.289 |  |
| Previous or ongoing drug treatments (n, %)  Antihypertensive drugs  Hypoglycemic drugs  Statins  Antiplatelet drugs | 121 (30.6)  95 (23.9)  51 (12.9)  83 (20.9) | 75 (25.8)  58 (19.9)  32 (11.0)  47 (16.2) | 0.201  0.225  0.398  0.124 |  |
